# Magnetic Properties of La_0.9_A_0.1_MnO_3_ (A: Li, Na, K) Nanopowders and Nanoceramics

**DOI:** 10.3390/ma13071788

**Published:** 2020-04-10

**Authors:** Paweł Głuchowski, Ruslan Nikonkov, Robert Tomala, Wiesław Stręk, Tatsiana Shulha, Maria Serdechnova, Mikhail Zheludkevich, Andrius Pakalaniškis, Ramūnas Skaudžius, Aivaras Kareiva, Alexander Abramov, Andrei Kholkin, Maxim V. Bushinsky, Dmitry Karpinsky

**Affiliations:** 1 Nanoceramics Inc., Okolna 2, PL-50422 Wroclaw, Poland; r.nikonkov@nanoceramics.pl; 2 Institute of Low Temperature and Structural Research PAS, Okolna 2, PL-50422 Wroclaw, Poland; r.tomala@intibs.pl (R.T.); w.strek@intibs.pl (W.S.); 3 Helmholtz-Zentrum Geesthacht, Zentrum für Material-und Küstenforschung GmbH, Max-Planck-Straße 1, DE-21502 Geesthacht, Germany; Tatsiana.Shulha@hzg.de (T.S.); maria.serdechnova@hzg.de (M.S.); mikhail.zheludkevich@hzg.de (M.Z.); 4 Christian-Albrechts-Universität Kiel, Christian-Albrechts-Platz 4, DE-24143 Kiel, Germany; 5 Vilnius University, Universiteto g. 3, LT-01513 Vilnius, Lithuania; pakalniskis.andrius@gmail.com (A.P.); ramunas.skaudzius@chgf.vu.lt (R.S.); aivaras.kareiva@chgf.vu.lt (A.K.); 6 School of Natural Sciences and Mathematics, Ural Federal University, Kuybysheva 48, RU-620026 Ekaterinburg, Russia; alexander.abramov@urfu.ru (A.A.); kholkin@ua.pt (A.K.); 7 CICECO-Materials Institute of Aveiro & Physics Dept, University of Aveiro, PT-3810-193 Aveiro, Portugal; 8 Scientific and Practical Materials Research Center NAS Belarus, P. Brovki 19, BY-220072 Minsk, Belarus; bushinsky@physics.by (M.V.B.); dmitry.karpinsky@gmail.com (D.K.); 9 South Ural State University, Prospekt Lenina, 76, RU-454080, Chelyabinsk, Russia

**Keywords:** multiferroic, manganites, alkali ions, ceramics, magnetization

## Abstract

Nanocrystalline La_0.9_A_0.1_MnO_3_ (where A is Li, Na, K) powders were synthesized by a combustion method. The powders used to prepare nanoceramics were fabricated via a high-temperature sintering method. The structure and morphology of all compounds were characterized by X-ray powder diffraction (XRD) and scanning electron microscopy (SEM). It was found that the size of the crystallites depended on the type of alkali ions used. The high-pressure sintering method kept the nanosized character of the grains in the ceramics, which had a significant impact on their physical properties. Magnetization studies were performed for both powder and ceramic samples in order to check the impact of the alkali ion dopants as well as the sintering pressure on the magnetization of the compounds. It was found that, by using different dopants, it was possible to strongly change the magnetic characteristics of the manganites.

## 1. Introduction

The discovery of the colossal magnetoresistance (CMR) effect in alkaline ion (A)-doped rare earth (RE) perovskite-type manganites with a composition of RE_1−x_A_x_MnO_3_, has attracted ample attention in recent years [1,2,3]. The studies on such manganites have revealed that the CMR effect is attributed to the presence of Mn^3+^ and Mn^4+^ ions together with the site–site double-exchange (DE) mechanism [4]. The DE mechanism links the electronic properties to the magnetic transition and describes the hopping of electrons in e_g_ orbitals between neighboring Mn^3+^ and Mn^4+^ ions with strong on-site Hund’s coupling by an O^2−^ anion. To induce the formation of Mn^4+^ ions, manganites are doped with either mono- [5,6] or divalent ions [7,8], which, in turn, leads to the compensation of the charge and formation of Mn^4+^ in the place of Mn^3+^. A lot of the studies suggest that the ground states of manganites are intrinsically inhomogeneous and characterized by the presence of competing phases extending over domains, especially at the nanoscale [9,10]. As the CMR effect is also often attributed to the Jahn–Teller (JT) effect [11], a parent compound of several important mixed-valence CMR manganite families, LaMnO_3_ (LMO), is at the focus of intensive investigations [12,13]. Double perovskite-type manganites with a general formula AA′BMnO_6_ (where A and A′ are the rare earth or alkaline earth metals, and B is the d-block transition metals) also display a wide variety of interesting physical properties based on their compositional variations [14,15,16]. The usage of different dopants and changing the chemical make-up of manganites is focused on obtaining a material that can be used at room temperature and in a relatively low magnetic field.

Magnetic studies of manganites are performed by many research groups on powders [17,18], single crystals [19], ceramics [20] or thin films. The form of the material is related to its future application, but it is also interesting to point out the fact that some materials, even with the same composition, may possess different properties. For example, Markovich et al. [21] observed that the magnetic properties of La_1−x_MnO_3+δ_ are dependent on the size of the nanocrystals. Meanwhile, Sacchetti et al. [22] observed that the Curie temperature (T_C_) in manganites is dependent on the applied pressure that activates localized antiferromagnetic (AF) interactions between the core spins. The effect of hydrostatic pressure on the electrical resistivity and on the ferromagnetic transition temperature in ceramic manganite was described by Garbarino et al. [23]. They found that the pressure effect is also dependent on the composition of the manganites.

Taking all of the above into account, nanocrystalline La_0.9_A_0.1_MnO_3_ (where A is Li, Na, K) powders were prepared to check the impact of the alkaline ion type on their magnetic properties. The aforementioned nanopowders were used to sinter ceramics under high pressure. Since for nanocrystalline powders, some of the properties remain even after the release of pressure [24], it was expected that the applied pressure would change the magnetic properties of nanomanganites permanently. To check the impact of the used dopants and applied pressure, structural and magnetic properties were investigated. It was found that both the alkaline ion used and the sintering pressure had a significant impact on the magnetic properties of La_0.9_A_0.1_MnO_3_.

## 2. Materials and Methods

To obtain the La_0.9_A_0.1_MnO_3_ compounds (where A is Li, Na, K), a combustion method was applied. Firstly, La(NO_3_)_3_•6H_2_O, Mn(NO_3_)_2_•4H_2_O and a precursor (LiNO_3_, NaNO_3_ or KNO_3_) with a molar ratio of La/A = 0.9/0.1 were added into an excess of 10% molten stearic acid in a porcelain reactor. For the next step, the resulting mixture was continuously stirred and kept at 140 °C for a sufficient period of time to allow the La-Mn-stearic acid gel to be formed (around 2 h). Then, the porcelain crucible reactor was heated up to 500 °C. At this stage, the gel volatilized and auto-ignited, with the evolution of a large volume of gases in turn producing a loose powder. The synthesis of the powder in the first stage was run under reducing conditions that may have led to a deficiency of oxygen in the atmosphere. Therefore, the obtained loose powders were calcined at 700 °C for 1 h in air atmosphere. All powders were ground in an agate mortar after calcination and then used for further experiments. Part of the powders was sintered into ceramics using a high-pressure sintering technique [25]. Briefly, the powders were formed into pellets and placed in a specially shaped container with a graphite heater and separated by boron nitride layers. The force exerted by the anvils produced a quasi-isostatic pressure of 8 GPa and the pellet was sintered at 500 °C in air. The pellets were heated at a rate of 10 °C/s to 500 °C and then sintered at this temperature for 1 min. The cooling took about 1 min. The ceramics were then removed from the container and polished using a grinding paper.

Structural studies were performed by powder X-ray diffraction (XRD) using a PANalytical X’Pert diffractometer (Malvern Panalytical, Almelo, The Netherlands) with Ni-filtered CuK_α_ radiation, λ = 0.15418 nm. Scanning electron microscopy (SEM) was used to inspect the grain size as well as the morphology of both the powders and ceramics. SEM characterization of the powders was done using a commercial Tescan Vega3 SB microscope (SEM, Brno, Czech Republic). The compositional homogeneity of the powders was checked using an EumeX EDX analyzer (Heidenrod, Germany). SEM micrographs of the ceramics were taken using a Merlin field emission scanning electron microscope (Carl Zeiss NTS, Germany). Isothermal magnetization measurements were performed in magnetic fields up to ±14 T at 5 and 300 K, and temperature dependencies of magnetization were measured in zero-field-cooled (ZFC) and field-cooled (FC) modes within an applied magnetic field of 1 kOe using Physical Properties Measurement Systems from Cryogenic Ltd. (London, UK).

## 3. Results

### 3.1. Structure and Morphology

**Figure 1:**
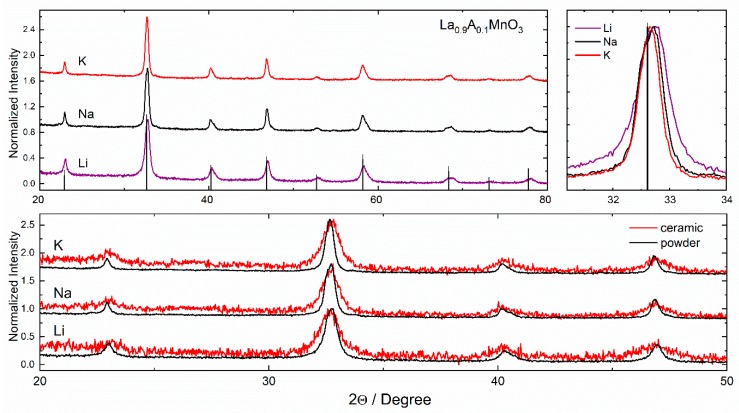
XRD patterns measured for La_0.9_A_0.1_MnO_3_ powders and ceramics.

The La_0.9_A_0.1_MnO_3_ powders and ceramics were characterized by X-ray diffraction performed at room temperature (Figure 1). The crystal structure and the unit cell parameters were calculated by Rietveld refinement using X’pert HighScore Plus software. This procedure revealed that the crystal structure of the manganite nanoparticles was rhombohedral with an R-3c space group. Further, it was observed that the diffraction peaks became narrower and shifted towards lower 2Theta values when the ionic radius of the dopant increased (Li: 0.76 Å, Na: 1.02 Å and K: 1.38 Å with CN = 6). This suggests that with an increase in the ionic radius of the dopant, replacing La ions (1.032 Å), the lattice expands. As in the case for the lattice parameters, we also observed an increase in the cell volume. The gradual increase in the unit cell parameters with the increase in the ionic radius of the dopant elements confirmed a formation of single-phase solid solutions. It should be noted that a larger ionic radius of the dopant ion resulted in a reduction in the structural distortion of the compounds, thus the ratio of the reduced unit cell parameters c/a became closest to a ratio for the compounds with larger dopant ions.

The Rietveld method using the X’pert Pro software was also employed in order to refine the ionic position coordinates. The atomic site positions that are compatible with the R-3c space group were considered during calculation as follows: La (0, 0, 0.25) in 6a, Mn (0, 0, 0) in 6b and O (x, 0, 0.25) in 18e, where x ≈ 0.46. It should also be noted that the accuracy of the refinement was greatly affected by uneven grain size distribution and broadening of the diffraction peaks caused by internal strains in the nanostructures and a rather small crystallite size. All of the obtained results are presented in Table 1. From the obtained data, a much higher cell expansion and increase in strain for the ceramics was observed as compared to powders. This was related to the mechanical stress that was applied during the sintering of the ceramics. The difference between the powders and ceramics may be explained by taking the size effect into consideration. In the case of powder, this effect is related only to the increase in the ion radius leading to the increase in the unit cell volume and elongation of the bonds between the ions. In the case of ceramics, the core–shell structure of the particles should also be considered. The bonds between anions and cations are different in the surface layer and inside the grains. As a result, after applying critical pressure, the surface of the particles transforms to the amorphous state that belongs to the shell. The “shell” phase can be detected by XRD as a relaxed cell unit with a higher volume [26]. Therefore, the expansion of the unit cell is higher for ceramics than for powders.

**Figure 2:**
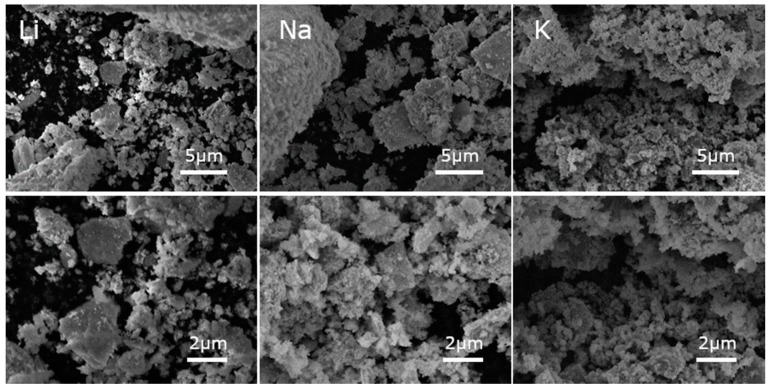
SEM micrographs of La_0.9_A_0.1_MnO_3_ (A: Li—**left**, Na—**center**, K—**right**) powders.

**Figure 3:**
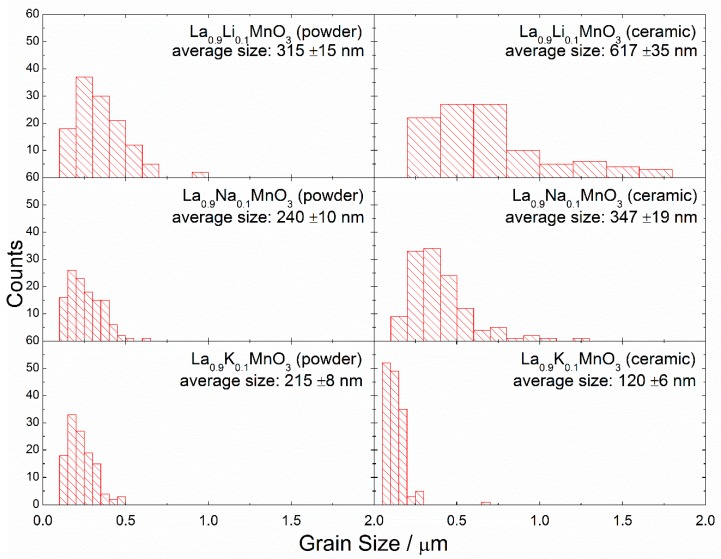
Distribution of the grain sizes in the La_0.9_A_0.1_MnO_3_ (A: Li, Na, K) nanopowders and nanoceramics.

The SEM micrographs taken for La_0.9_A_0.1_MnO_3_ (where A is Li, Na, K) show that the prepared powders were composed of microsized aggregates of nanosized particles (Figure 2). It can be noticed that, when the size of the alkali ion dopant increased, the grains became smaller. The particle sizes were estimated from SEM micrographs using ImageJ software [27]. The size of single particles were estimated to be around 315 ± 15, 240 ± 10, and 215 ± 8 nm for Li-, Na- and K-doped powders, respectively, and for ceramics 617 ± 35, 347 ± 19, and 120 ± 6 nm, respectively (Figure 3). For powders doped with smaller alkali ions, growth of the grains after sintering was observed, but in the case of potassium the situation was reversed. This may be the result of two phenomena. First, the bigger ion may have expanded the cell volume, increasing the distance between the ions. Second, the applied pressure may have caused the decomposition of the grain surface and this effect may be stronger in case of powder, where the distances between ions are longer. The decrease in the grain size in nanoceramics after the application of pressure was observed in our previous paper [28], and it was related to the surface amorphization.

**Figure 4:**
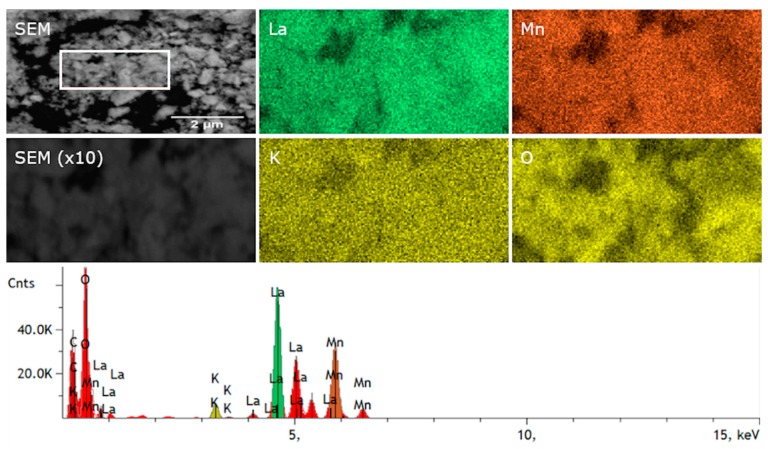
SEM and EDS map of La_0.9_K_0.1_MnO_3_ powder.

The compositional maps of SEM/EDS (Energy Dispersive Spectroscopy) micrographs show the main elements (La, Mn, K and O) and their distribution in the La_0.9_K_0.1_MnO_3_ powder (Figure 4). All of the elements were homogeneously distributed without any significant aggregation in grain boundaries. According to the compositional analysis (Table 2), the La_0.9_K_0.1_MnO_3_ powder contained 22.2 mol% of O, 1.08 mol% of K, 9.52 mol% La and 10.62 mol% of Mn (C came from SEM carbon tape), all of this was in good agreement with the designed ratio of the cations. The low content of the oxygen in the EDS analysis may have been caused by the low sensitivity of the EDS on lighter elements. Most EDS software will quantify the content of oxygen by stoichiometry of the compound, which is typically more accurate when the valence states of the cations are known. We predicted that in the samples there coexisted an Mn^3+^ and an Mn^4+^ cation, and therefore the stoichiometry may have been distorted, which in turn resulted in inaccuracies in the EDS analysis. An excess or deficient oxygen in the rhombohedral structure of manganites could also have manifested as the splitting of diffraction peaks in XRD measurements [29]. As shown in Figure 1, no splitting was observed, which confirmed the stoichiometric amount of oxygen in the compounds.

**Figure 5:**
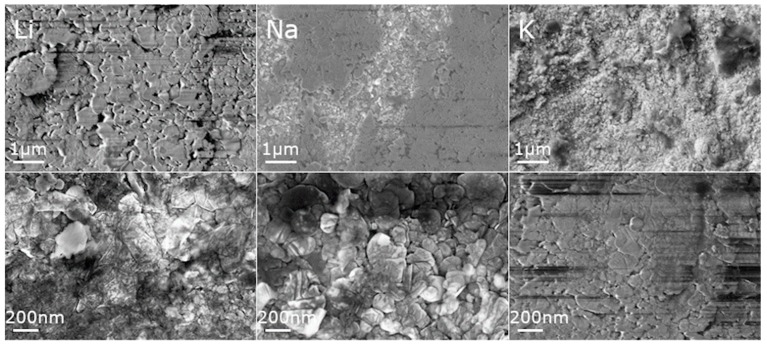
SEM micrographs of La_0.9_A_0.1_MnO_3_ (A: Li—**left**, Na—**center**, K—**right**) ceramics.

SEM micrographs of the ceramics were taken to check the impact of the applied pressure on the morphology of the nanocrystals after the sintering procedure. It can be seen that after sintering, the grains decreased in size (Figure 5). For all ceramics, pores were observed, although for the smallest particles (K-doped), the number of them was strongly reduced.

### 3.2. Magnetic Properties of Powders and Ceramics

Temperature-dependent and isothermal field magnetization measurements were performed for the compounds in the temperature range of 5–300 K. They clarified the evolution of the magnetic structure as a function of the dopant element and temperature. The magnetization data confirmed the presence of a strong ferromagnetic component, which was specific for all of the compounds under examination regardless of their form, ceramic or powder.

**Figure 6:**
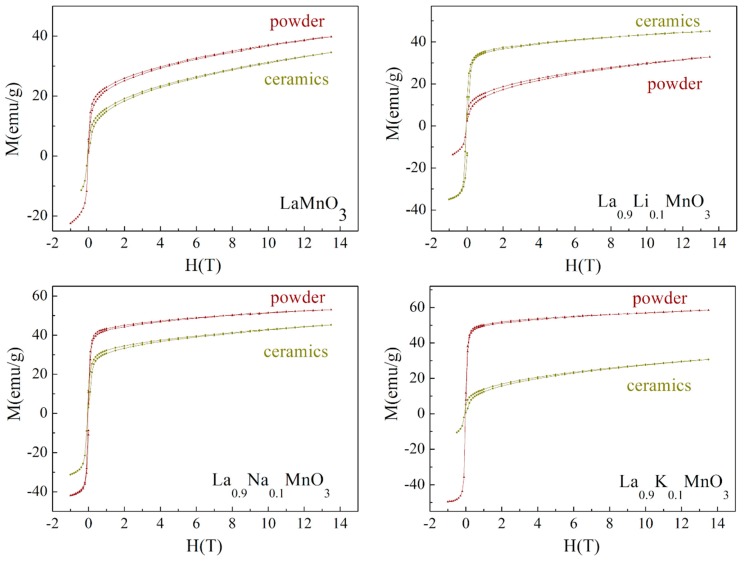
Isothermal magnetization curves measured for the initial compound LaMnO_3_ and doped La_0.9_A_0.1_MnO_3_ (A: Li—**right top**, Na—**left bottom**, K—**right bottom**) powder and ceramics at T = 5 K.

It is well known that the bulk stoichiometric compound LaMnO_3_ is characterized by the collinear antiferromagnetic state with a Neel temperature of ~140 K [30]. The isothermal magnetization data obtained for the initial compound affirmed its inhomogeneous magnetic state, which was most probably caused by a lack of the cation content, which is in accordance with the EDX data obtained for the compounds (Figure 6). Assuming the results of the magnetization measurements, one can confirm the coexistence of the ferromagnetic phase along with the antiferromagnetic component. The information about the crystal structure obtained by the X-ray diffraction measurements points at the rhombohedral distortion of the unit cell which was specific for the LaMnO_3+δ_ compounds having a nominal oxygen excess δ more than 1% [31]. It should be noted that the value of magnetization estimated for the LaMnO_3_ compound in powder form at 5 K can be justified, assuming that about 20% of the manganese ions were in a 4+ oxidation state, so the ferromagnetic component was caused by strong positive exchange of Mn^4+^ – O – Mn^3+^ interactions described by the double exchange mechanism [32]. Meanwhile, the negative exchange of Mn^3+^ – O – Mn^3+^ interactions formed a notable antiferromagnetic component. The value of magnetization obtained for the LaMnO_3_ compound in the form of ceramic in a magnetic field of 14 T was about 15% lower than that attributed to the compound in powder form. The difference could potentially have been caused by a more distinct oxidation than that which occurred for the compound in powder from, which correlates with the peculiarities of the preparation method.

Doping the compounds with alkali metals caused a notable modification of the magnetic state, while their crystal structure showed only minimal changes, remaining a rhombohedral type of lattice distortion. The chemical substitution of La^3+^ ions by monovalent Na or K ions caused an increase in the magnetization value which reached ~60 emu/g at a magnetic field of 14 T and spontaneous magnetization of ~50 emu/g for potassium-doped compound in powder form (Figure 6). Such parameters of magnetization are characteristic for alkali-earth-doped manganites having long-range ferromagnetic order and showing a maximal value of magnetization among the complex magnetic oxides with a perovskite structure [33,34]. It should be noted that chemical doping by Na or K ions causes an increase in the unit cell parameters accompanied with an elongation of the *c*-parameter of the rhombohedral unit cell, however, the associated modification in the Mn – O – Mn chemical bonds cannot justify the abovementioned changes in the magnetization. The alterations in the magnetic state noted for the doped compounds can be explained assuming the different ratio of Mn^4+^ / Mn^3+^ ions, which varies depending on the preparation conditions and redox properties of the initial reagents. Moreover, the magnetization value observed for the Li-doped compound in a powder form was lower than the value measured for the initial compound, which could have been caused by the lower amount of the Mn^4+^ ions in the doped compound, due to lower oxidation during the synthesis process.

**Figure 7:**
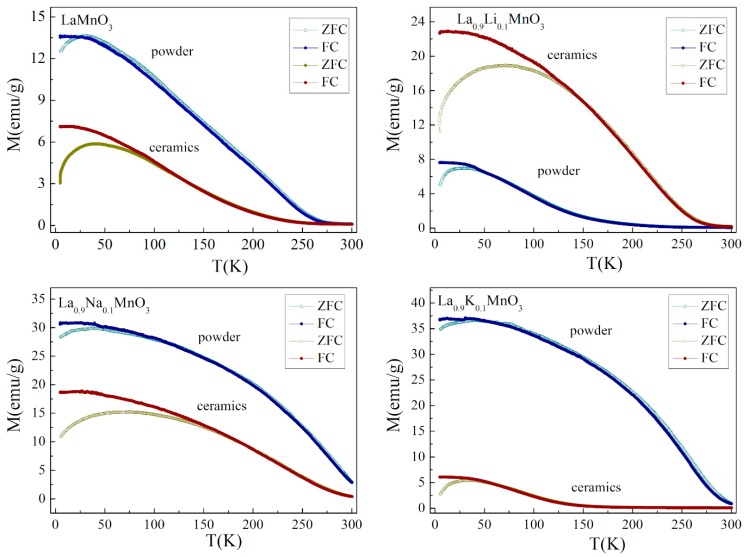
Field cooled and zero-field cooled dependencies of magnetization recorded for the La_0.9_A_0.1_MnO_3_ compounds (A = Na, Li, K) and initial compound LaMnO_3_ in a magnetic field of 1kOe.

Figure 7 shows the magnetization dependencies on temperature measured in zero-field-cooled (ZFC) and field-cooled (FC) modes under an applied magnetic field of 1kOe. Analysis of the M(H) and M(T) curves allowed the tracing of evolution of the magnetization as a function of the magnetic field, temperature and the type of dopant ions. The M(T) dependencies obtained for the initial compound LaMnO_3_ confirmed the presence of a long-range ferromagnetic-like order, which was attributed to the compounds in both powder and ceramic forms. Analysis of both field and temperature dependencies of magnetization pointed to the presence of a substantial antiferromagnetic component along with a long-range ferromagnetic phase. The ferromagnetic phase in the LaMnO_3_ compound was caused by the positive exchange Mn^3+^ – O – Mn^4+^ interactions while the antiferromagnetic component was determined by the negative exchange interactions formed between homovalent manganese ions in a 3+ oxidation state. It should be noted that stoichiometric LaMnO_3_ is characterized by the dominant antiferromagnetic phase caused by negative exchange Mn^3+^ – O – Mn^3+^ interactions [32,33]. The value of spontaneous magnetization estimated for the compound in powder form was nearly double that attributed to the ceramic compound, while the temperature of the transition to a non-magnetic state was nearly equal for both compounds. The difference in the magnetization was caused by more active oxidation processes that occurred in nanoscale grains of the compound in a powder form, which resulted in a larger ratio of Mn^4+^/Mn^3+^ ions in the powder compound as compared to that estimated for the ceramic compound. A small divergence in the M(T) curves measured in FC and ZFC modes occurred only at low temperatures, which is in accordance with the small coercivity observed for the field dependencies of magnetization.

The M(T) curves obtained for the compounds doped with Na ions attested to the presence of a strong ferromagnetic component in both the ceramic and powder compounds. The spontaneous magnetic moments calculated for the sodium-doped compounds at a temperature of 5 K (M_S_~1.85 μ_B_ and ~1.45 μ_B_ for powder and ceramic compounds, respectively) was nearly twice as high as the magnetic moments estimated for the initial LaMnO_3_ compound, and the obtained difference was in accordance with the ratio Mn^4+^/Mn^3+^ determined by the chemical doping. A substitution of 10% La ions with monovalent Na ions led to an increase in the amount of Mn^4+^ ions up to about 40%. Potassium-doped compounds were characterized by the more prominent difference in the magnetization data obtained for the samples in powder and ceramic form. The magnetization measurements performed for the compound in powder form demonstrated the presence of a long-range ferromagnetic phase with a magnetic transition temperature of ~280 K; while magnetization data obtained for the compound in ceramic form showed cluster glass behavior with a blocking temperature of ~40 K. The most feasible model to describe the magnetic properties of the ceramic La_0.9_K_0.1_MnO_3_ compound assumes the coexistence of both ferro- and antiferromagnetic phases similar to the situation observed for the initial LaMnO_3_ compound. The difference in magnetic properties observed for the potassium-doped compounds in powder and ceramic forms was caused by a significant amount of Mn^4+^ ions formed in the powder compound due to the active oxidation process that occurred in the nanoscale grains of the sample which led to the formation of long-range ferromagnetic order with a spontaneous magnetic moment of ~2.15 μ_B_ at 5 K. On the other hand, the small average grain size estimated for the K-doped LaMnO_3_ ceramics (~120 nm) was associated with a reduction in long-range magnetic order, as the compound contained about 30% of the grains with a characteristic size of about 50 nm (see Table 2). It is known that a decrease in the grain size below 50 nm leads to a drastic reduction in the long-range magnetic order [35,36], and this certainly causes a reduction in the magnetization of the compound. Lithium-doped compounds were the only compounds under study, which demonstrated a magnitude of magnetization in the ceramic compound larger than that calculated for the powder compound. The spontaneous magnetic moment calculated for the lithium-doped compound in ceramic form was ~1.5 μ_B_ at 5 K, which was the maximal value among the magnetic moments observed for the ceramic compounds under study. On the other hand, the spontaneous magnetic moment of the compound in powder form was nearly a half of the magnetic moment calculated for the compound in ceramic form. An unexpectedly small magnetization observed for the powder compound was most probably associated with inert oxidation processes that occurred in the compound during the synthesis procedure.

## 4. Conclusions

A series of La_0.9_A_0.1_MnO_3_ nanopowders doped with Li, Na and K ions were successfully synthesized via combustion method. The as-obtained powders were used to prepare ceramics with the help of a high-pressure sintering method. It was found that, when the size of the dopant ion increased, the grains of manganites became smaller. The sintering process led to an increase in the grain size in the case of small radius doping ions (twice for Li, slightly for Na) and a decrease in grain size in the case of the K dopant. It was found that the type of alkali ion and the structure (powder vs. ceramic), as well as the size of the grains, have a significant impact on the magnetic properties of these manganites. Magnetization measurements performed on the compounds clarified their magnetic property dependencies, in a wide range of temperatures and magnetic fields, depending on the dopant type and grain morphology. The results of the magnetization measurements obtained for the initial LaMnO_3_ compound point at its inhomogeneous magnetic state caused by the coexistence of the ferromagnetic phase along with the antiferromagnetic component. The ferromagnetic phase of the compounds was caused by the positive exchange interactions between heterovalent manganese Mn^3+^ – O – Mn^4+^ ions formed due to an active oxidation process during the sample preparation, while the antiferromagnetic component was caused by the negative exchange interactions between homovalent Mn^3+^ ions. The chemical substitution of La ions by monovalent alkali elements caused an increase in the amount of Mn ions having a 4+ oxidation state and thus an increase in the magnetization. The doped compounds demonstrated complex magnetic behavior caused by the competing ferromagnetic and antiferromagnetic components, which were determined by the activity of the oxidation processes occurring during the sample preparation as well as the size and morphology of the crystallites. It should be noted that the possibility of controlling the magnetic properties of the compounds, viz. magnetization, coercivity and magnetic transition temperature, increases the attractiveness of the use of the studied manganites in various technological applications.

## Figures and Tables

**Table 1:** Cell parameters obtained from XRD patterns for La_0.9_A_0.1_MnO_3_ powders and ceramics.

	a, b	c	V	Strains	Density
	Å	Å	Å^3^	%	g/cm^3^
Powders					
La_0.9_Li_0.1_MnO_3_	5.4974	13.3149	348.49 (58.08)	0.073	6.62
La_0.9_Na_0.1_MnO_3_	5.5017	13.3382	349.65 (58.27)	0.051	6.60
La_0.9_K_0.1_MnO_3_	5.5044	13.3693	350.79 (58.47)	0.046	6.58
Ceramics					
La_0.9_Li_0.1_MnO_3_	5.4979	13.3198	348.68 (58.11)	0.136	6.91
La_0.9_Na_0.1_MnO_3_	5.5080	13.3402	350.51 (58.42)	0.094	6.86
La_0.9_K_0.1_MnO_3_	5.5088	13.3798	351.64 (58.61)	0.083	6.82

**Table 2:** EDS spectrum analysis of La_0.9_K_0.1_MnO_3_ powder.

Element	Line	Intensity	Concentration	Concentration	Error
		(c/s)	wt.%	mol%	2-sig
C	Ka	96.28	7.79	-	0.114
O	Ka	196.26	14.21	22.2	0.148
K	Ka	24.95	1.69	1.08	0.068
Mn	Ka	132	23.37	10.62	0.287
La	La	228.79	52.94	9.52	0.401
Total			100		
